# Clinical evaluation of three deep learning contouring models for head and neck organs at risk and lymph node levels

**DOI:** 10.1016/j.tipsro.2026.100437

**Published:** 2026-09-23

**Authors:** Adam Miovecz, Bettina Nagy, Daniel Gugyeras, Daniel Fajtai, David Sipos, Csaba Vandulek, Arpad Kovacs, Ferenc Lakosi

**Affiliations:** a Department of Medical Imaging, Faculty of Health Sciences, University of Pecs, Kaposvar, 7400, Hungary; b Doctoral School of Health Sciences, Faculty of Health Sciences, University of Pecs, Pecs, 7621, Hungary; c Department of Radiation Oncology, Dr. Jozsef Baka Center, Moritz Kaposi General Hospital, Kaposvar, 7400, Hungary; d Medicopus Non Profit Ltd., Kaposvar, Hungary; e Department of Oncoradiology, Faculty of Medicine, University of Debrecen, Debrecen, 4032, Hungary

**Keywords:** AI, Deep Learning, H&N, Lymph node

## Abstract

**Introduction:**

Our aim was to compare the accuracy and clinical acceptability of head and neck organs at risk (OAR) and lymph node contours generated by three commercially available deep learning contouring (DLC) models: Mirada DLCExpert (Model D), Limbus AI (Model L), and MVision AI Contour+ (Model C).

**Methods:**

Seven OARs and cervical lymph node levels I to V were delineated by each model on twenty retrospectively selected patients with hypopharyngeal/laryngeal tumors (2020 to 2023). Automated contours were compared against expert reference contours using the Dice similarity coefficient (DSC), the 95th percentile Hausdorff distance (HD95), the median surface distance (MDSD) and the Jaccard index. Differences among the three models were tested with the Friedman test followed by pairwise Wilcoxon signed-rank tests with Holm-Bonferroni correction; agreement of structure volumes with the expert reference was tested with the Wilcoxon signed-rank test. A structured qualitative assessment classified each contour as requiring major corrections, minor corrections, or acceptable as is. A sensitivity analysis repeated every comparison using only reference contours independent of all three models.

**Results:**

Pooled over the seven OARs, mean DSC was 0.85 for Models L and C and 0.80 for Model D, but this ordering is produced entirely by the esophagus and the spinal canal, for which Model D applies a different anatomical definition (mean DSC 0.40 and 0.57, with model volumes 35% and 44% of the expert volume). Across the other five OARs Model D was the most accurate (0.92 against 0.84 and 0.85). After Holm-Bonferroni correction, Models L and C did not differ on any structure or metric. For lymph node levels no overlap metric separated the models in the primary analysis (all *p* > 0.05), though the omnibus test became significant in favour of Model D when restricted to reference contours independent of the models; Model D had the highest mean DSC (0.84) and the widest spread and Model C much the narrowest. Model C was the only model whose lymph node volumes showed no systematic difference from the expert reference (260.4 vs 256.6 cm3, mean difference + 3.8 cm3, 95% limits of agreement −46.5 to +54.1 cm3), against −33.4 and + 30.5 cm3 for Models D and L. Manual contouring averaged 28 min (OARs) and 16 min (lymph nodes); AI-assisted review took approximately 5 min per patient in total. In the subjective assessment Models L and C required fewer corrections than Model D (mean modification score 2.44 and 2.43 vs 2.03) and were comparable to each other.

**Conclusion:**

All three models produced contours of clinically usable quality apart from Model D on the esophagus and the spinal canal. Models L and C were not statistically different throughout. Model D differed from both on the esophagus and the spinal canal, where it applies a narrower anatomical definition, and was the most accurate model on the remaining five OARs, though that advantage was only partly confirmed when the analysis was restricted to reference contours independent of the models. Model C gave the most predictable lymph node output: the narrowest spread and the only volumes not differing significantly from the expert reference. Deep learning contouring models significantly improve head and neck treatment planning efficiency by providing clinically acceptable accuracy in a fraction of the time required for manual contouring.

## Highlights

• Three commercial DLC models compared for H&N OARs and node levels I-V (*n* = 20).
• Models L and C did not differ on any structure or metric after correction.
• Model D's low scores reflect a structure-definition mismatch, not poor segmentation.
• Only Model C matched expert lymph node volume (bias +3.8 cm3, *p* = 0.70).
• AI-assisted review cut delineation from 44 min to about 5 min per patient.

## Introduction

Advances in Intensity Modulated Radiation Therapy (IMRT), Volumetric Modulated Arc Therapy (VMAT), and hybrid imaging (PET/CT, PET/MR) have enabled increasingly precise radiation delivery in the head and neck, with higher resolution imaging facilitating more detailed contouring [1], [2].

Accurate delineation of anatomical structures and lymph node levels is essential for effective treatment planning and side effects minimization, however manual contouring remains labor intensive (20 to 40 min per patient) and prone to inter-and intra-observer variability [3], [4], [5], [6].

This variability is particularly pronounced for cervical lymph node levels, where soft tissue boundaries are inherently ambiguous. In a STAPLE based analysis, Balik et al. reported contour volumes ranging from 106 to 430 cm^3^ across five physicians for the same patient [7] while van der Veen et al. showed that deep learning based delineation reduced the inter-observer median surface distance (MDSD) from 2.5 to 1.4 mm and correction times from 52 to 35 min [8]. Pang et al. (2025) confirmed these findings across seven global institutions [9]. A recent systematic review reported that Deep Learning Contouring (DLC) edited contours frequently achieve a mean Dice Similarity Coefficient (DSC) above 0.85 for target volumes and above 0.90 for well-defined Organs at Risk (OAR) [10].

Atlas based contouring was the first major step in automated segmentation, relying on preexisting anatomically labeled images to propagate contours onto new patient scans. Its main advantages over manual contouring were reduced contouring time and inter-observer variability [11]. Advances in artificial intelligence (AI) and deep learning (DL) have since introduced a new paradigm for automated contouring, offering the potential to further improve consistency, quality, and efficiency in the radiotherapy workflow [12]. DLC based auto segmentation, using multi-layer neural network models, can be completed within one minute compared to over 20 min for manual segmentation [13], with reported reductions of up to 76% in total contouring time [14]. Studies have shown that DLC contours for structures like the brainstem and parotid glands exhibit less variability compared to manual contours [1], [15].

The performance of DLC models depends on the quality and quantity of training data [16]. Despite promising results, integration into clinical practice requires overcoming trust issues among clinicians and physicists and ensuring that models can handle variability in clinical data. DLC can also facilitate adaptive radiotherapy by rapidly updating contours based on anatomical changes during the treatment course [17]. Developing and adhering to consensus guidelines for OAR delineation can further enhance model reliability [6], while implementing robust quality assurance protocols is essential for validating DLC generated contours before clinical use [18].

Recent multi-vendor evaluations have compared commercial DLC platforms for head and neck OAR contouring [19], however data on lymph node level segmentation across vendors remains scarce. This study addresses that gap by evaluating the geometric accuracy and clinical acceptability of contours from three commercially available DLC models for both head and neck OARs and lymph node levels, using quantitative metrics and expert assessment.

## Materials and methods

### Patients

Twenty patients with hypopharyngeal or laryngeal tumors were randomly selected from a retrospective cohort of 144 patients treated at XXX between 2020 and 2023. No patients were excluded based on tumor stage or minor anatomical variations to ensure the DLC models were evaluated against a realistic, heterogeneous clinical workload; however, patients with a history of prior major reconstructive surgery or neck dissection altering the standard bilateral nodal levels were excluded. Seventeen patients received definitive radiotherapy (60 to 70 Gy in 21 to 35 fractions); three patients received palliative treatment (20 Gy in 4 to 5 fractions). Planning CTs were acquired on a Siemens SOMATOM Definition AS+ (3 mm slice thickness, 120 kV) with standard immobilization.

The present retrospective study was approved and conducted in accordance with the institutional research and ethics guidelines (135-PTEREKEB2026/1) and with the Helsinki Declaration of 1975 and 1989.

Furthermore, prior to analysis, all patient data were fully anonymized in accordance with the Hungarian GDPR regulation (Act XLVII of 1997 on the Processing and Protection of Health Data and Related Personal Data) and the GDPR Regulation (EU) 2016/679.

### Auto-segmentation process

Three commercially available DLC platforms were evaluated: Mirada DLCExpert (Model D), Limbus AI (Model L) and MVision AI Contour+ (Model C). Each platform was used with its default head and neck model and its vendor-supplied configuration; no site-specific retraining or model adaptation was performed. The three platforms are described in detail below.

The following OARs were evaluated: spinal canal, bilateral parotid glands, esophagus, bilateral submandibular glands, and brainstem. This set represents the common OARs supported by all three platforms; other structures available in individual models were excluded to enable direct comparison. Similarly, only lymph node levels I to V were analyzed, as these represent the shared anatomical scope across all three models.

Cervical lymph node levels I to V were contoured as separate structures for planning but were merged into a single lymph node region of interest before metric computation, because the three platforms do not use a common level-by-level nomenclature. All lymph node results reported here therefore refer to that combined region; per-level metrics are not available.

In the present study, ‘expert’ is defined as a senior radiation oncologist with extensive clinical experience at our institution; all Expert contours were delineated in strict accordance with ESTRO guidelines. [6]

For each patient a combined structure set was assembled, holding the three model contour sets under a model-specific naming convention together with the expert reference set, and in which the common starting slice was defined. The expert reference contours were delineated by the senior radiation oncologist following the ESTRO/DAHANCA/EORTC consensus guidelines, with the display of all automatically generated contours switched off; the contours of the original clinical treatment plans were not used as reference. Because the reference contours were held in the same structure set as the model output rather than in a separate one, the reference set is not structurally independent of the models by construction, and the archived data do not allow the order in which the four contour sets entered each structure set to be established: across the twenty patients the expert block precedes the Limbus and MVision blocks in every case and the Mirada block in fourteen of twenty, but ROI numbering is reassigned on import and copy operations and therefore does not record the delineation chronology. On re-analysis of the per-patient data, 49 of the 476 structure-patient comparisons (10.3%) were found to be effectively identical to one of the automatically generated contours. The criterion applied was DSC ≥ 0.99; in 48 of the 49 the HD95 was also exactly 0.0 mm, in all 49 the MDSD was 0.0 mm, and in all 49 the model and reference volumes agreed to better than 1% (median 0.18%). These 49 comparisons involve seventeen of the twenty patients and all three models (Model D 28 structures, Model C 12, Model L 9). In each case the reference contour and the model contour are two distinct ROIs carrying identical contour data, so the identity is a property of the structure sets themselves rather than of the comparison software; the mechanism by which it arose could not be reconstructed from the archived data. The primary analysis below retains every patient; a pre-specified sensitivity analysis restricted to reference contours that are independent of all three models is reported in Supplementary Table S3. The affected contours are listed per patient in Supplementary Table S4 and the supporting distributions shown in Supplementary Fig. S1.The full workflow is summarised in Fig. 1.

**Fig. 1:**
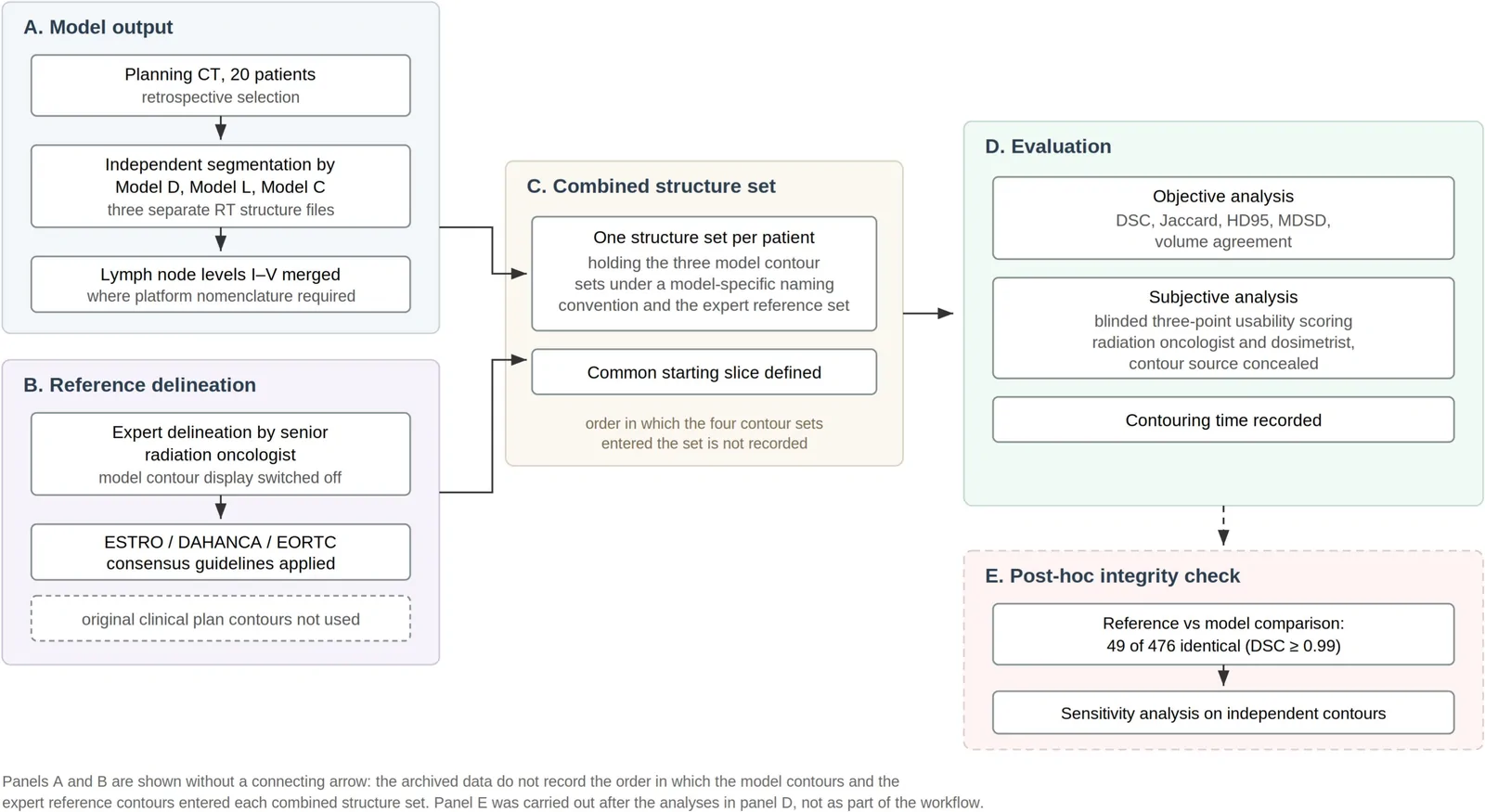
Workflow of the objective and subjective evaluation. Panels A and B are shown without a connecting arrow: the archived data do not record the order in which the model contours and the expert reference contours entered each combined structure set. Panel E was carried out after the analyses in panel D and was not part of the planned workflow.

The three platforms differ in the training data and in the delineation conventions on which they are based, that is, in the anatomical rules defining where a structure begins and ends, and the vendors do not disclose these to the same level of detail. Model C documents adherence to published consensus guidelines in its technical documentation; for Models D and L the training data are described only in general terms in the publicly available documentation. All expert reference contours in the present study followed the ESTRO/DAHANCA/EORTC consensus guidelines, so any difference in delineation convention between a model and those guidelines contributes to the discrepancies reported here. This is addressed in the Limitations.

**Model D** (Mirada DLCExpert, Mirada Medical Ltd., UK): This is a locally deployed deep learning platform that utilizes multiple convolutional neural networks (CNNs) for voxel-wise labeling of planning CT images. The vendor states that the models are trained on expert-delineated datasets; the training data are not described in further detail in the public documentation (see below). Planning CT datasets were transferred via a preset DICOM pathway to the local Mirada network server for fully automated segmentation. [20] The automatically generated structure sets were returned to the treatment planning system (Varian Eclipse) over the same preset DICOM pathway, without any manual editing, and were subsequently exported from Eclipse as DICOM RT structure sets for analysis.

**Model L** (Limbus AI, v1.5.0, Limbus AI Inc., Canada): This is a deep learning platform utilizing a U-net-based architecture. The software automatically determines the appropriate acquisition protocol directly from the incoming DICOM metadata, and the corresponding deep learning model automatically generates the contours. The workflow was processed via a secure, on-premise Limbus server connected to the local network [21]. The generated structure sets were returned to the treatment planning system (Varian Eclipse) via DICOM, without any manual editing, and were subsequently exported as DICOM RT structure sets for analysis.

**Model C** (MVision AI Contour+, MVision AI, Helsinki, Finland): Cloud hosted deep learning platform. Models are mapped to CT acquisition protocol through a web-based configuration interface. The underlying model architecture is not publicly disclosed. Planning CT series were sent to the cloud platform over an encrypted DICOM connection; the returned structure sets were imported into the treatment planning system (Varian Eclipse) without any manual editing and were subsequently exported as DICOM RT structure sets for analysis.

The automatically generated contours from each model were evaluated against those manually delineated by the expert radiation oncologist. Both expert and auto contours were exported after anonymization as DICOM RT structure files and transferred to 3D Slicer for evaluation.

### Evaluation metrics

Automatically generated contours were compared against expert references using the following metrics:

**Dice Similarity Coefficient (DSC)** measures volumetric overlap between automatic (A) and manual (B) contours: DSC (A, B) = 2|A∩B| / (|A| + |B|). Values range from 0 (no overlap) to 1 (perfect agreement).

**95th Percentile Hausdorff Distance (HD95)** measures the distance (mm) at the 95th percentile of surface to surface distances between two contour sets, providing a robust measure of worst case disagreement while excluding outliers [1].

**Jaccard Index (J)** measures overlap relative to the union: J(A, B) = |A∩B| / |A∪B|.

**Median Surface Distance (MDSD)** is the median value of all surface to surface distances between segmentation and reference.

HD95 and MDSD were computed on the 1 mm voxel lattice without sub-voxel interpolation, so both are quantized: the attainable values are square roots of integers and 89% of the observed MDSD values are exactly 0.0 or 1.0 mm. MDSD should therefore be read as an indicator of whether the median surface voxel coincides with the reference rather than as a continuously resolved distance.

As an aid to interpretation, a DSC above 0.80 is conventionally treated as indicating clinically usable agreement for head and neck OARs and a DSC above 0.90 as agreement comparable to the difference between two human observers, while an HD95 of 2 to 3 mm is of the order of a single 3 mm CT slice. These conventions should be read with caution: geometric indices alone correlate only loosely with the editing effort a contour actually demands and with clinical acceptability, and surface-based measures such as added path length track correction time more closely. [22], [23]

Contour evaluation was performed in 3D Slicer (version 5.6.2). After installation of the SlicerRT extension, a custom Python script was executed in the Python console to automate the comparison. The script loads the expert reference structure set and the three automated structure sets for a given patient, maps the differing structure nomenclature used by the three vendors onto a common set of labels, and then computes DSC, Jaccard index, HD95 and MDSD for every corresponding structure pair in a single batch, writing the results to a spreadsheet. This removes the need to select each structure pair manually in the graphical interface, which was the rate-limiting step of the evaluation, and it eliminates the associated risk of mis-pairing structures between models.

### Subjective analysis

The assessment used a blind approach to reduce evaluator bias. A summary spreadsheet was created in which each OAR contour was independently evaluated per patient by the same experienced radiation oncologist who delineated the baseline reference structures (ensuring intra-observer consistency) and by one dosimetrist, a BSc-certified radiographer with fourteen years of experience in treatment planning and contouring, who is likewise regarded as an expert observer in this context. Both evaluators were completely blinded to the identity and source of the contours during the review process. Each structure was classified using a three-point scale, applied with the following predefined criteria:

a) Major corrections required: the contour could not be used as a starting point without substantial re-delineation, either because of a systematic error (incorrect craniocaudal extent, omission of part of the structure, or inclusion of an adjacent structure) or because editing was needed on more than approximately one third of the slices on which the structure appeared, with a potential dosimetric consequence.
b) Minor corrections required: the contour was usable as a starting point and required only local editing, typically on fewer than approximately one third of the slices, with no change expected to be dosimetrically relevant.
c) Acceptable as is: no editing required, or editing limited to changes the observer would not have made in routine clinical practice.

Each of the seven OARs was scored for each of the twenty patients and each of the three models by both observers, giving 420 assessments per observer. For quantitative summary the ordinal scale was converted to a numerical score (a = 1, b = 2, c = 3), so that a higher mean score denotes a contour requiring less editing, and a mean modification score was computed for each model, structure and observer. The two observers were not asked to reach a consensus; their scores are reported separately because they represent two distinct professional perspectives on clinical usability. Manual contouring time per patient was also recorded for both OARs and lymph node regions.

### Statistical analysis

Statistical analyses were performed to test, for each structure, whether the contours produced by the three DLC models differed from one another in their agreement with the expert reference. Agreement with the reference was quantified by four geometric endpoints and, separately, by structure volume. DSC and the Jaccard index quantify volumetric overlap and are therefore sensitive to agreement over the bulk of the structure; the two are related by J = DSC / (2 - DSC), so under rank-based testing they carry the same information and yield identical omnibus *p*-values. The Jaccard index is reported only for comparability with the literature. HD95 characterizes the worst-case boundary disagreement while suppressing the influence of isolated outlier voxels, and is the metric most closely related to the local editing effort demanded of the observer. MDSD summarizes the typical, non-outlier surface displacement. Absolute structure volume was analyzed separately because two contours can reach an identical DSC while differing systematically in size, a discrepancy that overlap metrics cannot reveal but that is directly relevant to dosimetry and to the direction of the manual correction.

Because the three models were applied to the same twenty patients, all comparisons are paired. With *n* = 20, and with the metrics either bounded (DSC, Jaccard) or strongly right-skewed (HD95, MDSD), normality could not be assumed, and non-parametric methods were used throughout. The Friedman test was used as the omnibus test for a difference among the three related models for a given structure and metric. Where the Friedman test indicated a difference, pairwise post-hoc comparisons (D vs L, D vs C, L vs C) were performed with the Wilcoxon signed-rank test and the resulting *p* values were corrected for the three comparisons per metric with the Holm-Bonferroni procedure, which controls the family-wise error rate and is uniformly more powerful than the classical Bonferroni correction. The exact null distribution of the Wilcoxon statistic was used rather than the normal approximation, because HD95 and MDSD are quantized on the voxel lattice and the approximation is unreliable in the presence of that many tied differences; for three MDSD comparisons the approximation returned p between 0.03 and 0.05 where the exact test returns p above 0.05. Correction was applied within each structure and metric and not across the 32 structure-by-metric families, so results with p between 0.02 and 0.05 should be read as exploratory. Analyses were performed in Python 3.12 with SciPy 1.17 and NumPy 2.4.

Agreement with the expert reference was assessed separately: for each model, the per-patient difference between the model and the expert lymph node volume was tested against zero with the Wilcoxon signed-rank test, so that a non-significant result indicates the absence of a systematic volumetric bias with respect to the expert. All tests were two-sided and significance was set at *p* < 0.05. Given the sample size, non-significant pairwise differences are reported as an absence of evidence of a difference and are not interpreted as evidence of equivalence, since the study was not designed or powered as an equivalence trial.

Because part of the reference set is not independent of the models (see above), the whole structure-by-structure analysis was repeated on the subset of patients whose expert contour for that structure matched no model at DSC ≥ 0.99. This sensitivity analysis is reported in Supplementary Table S3. Where the two analyses disagree, the sensitivity result is the more conservative and is the one quoted in the Discussion. Because HD95 and MDSD are quantized on the voxel lattice, the Wilcoxon signed-rank tests on these two metrics carry a substantial number of tied pairs; ties were handled by the Wilcoxon zero-difference procedure, and the resulting *p*-values should be read with that in mind.

## Results

### Organs at risk

Pooled across the seven OARs, mean DSC was 0.80 ± 0.23 for Model D, 0.85 ± 0.10 for Model L and 0.85 ± 0.13 for Model C (Table 1, Fig. 2). This pooled figure is a descriptive summary only: its spread is dominated by between-structure variation, and it is driven by two structures for which Model D applies a different anatomical definition (esophagus and spinal canal), for which Model D contours the spinal cord rather than the canal and a shorter cranio-caudal extent of the esophagus. Across the remaining five OARs - brainstem, both parotid glands and both submandibular glands - the ordering reverses: Model D reaches 0.92 ± 0.08 against 0.84 ± 0.11 for Model L and 0.85 ± 0.14 for Model C. Model D also showed a markedly wider distribution of values than the other two models across the OARs (Fig. 2). The parotid glands showed the highest agreement for Models D and L (DSC 0.87 to 0.94); for Model C the brainstem was marginally higher (0.88 against 0.87). The lowest mean DSC was Model D esophagus (0.40 ± 0.20), followed by Model D spinal canal (0.57 ± 0.14).

**Table 1:** Descriptive statistics (mean ± SD) for DSC, HD95, the Jaccard index and MDSD, by structure and model, against the expert reference.

**Structure**	**Metric**	**n**	**Model D**	**Model L**	**Model C**
Brainstem	DSC	20	0.910 ± 0.062	0.869 ± 0.051	0.879 ± 0.046
	HD95 (mm)	20	3.62 ± 1.70	3.25 ± 1.32	2.77 ± 1.07
	Jaccard	20	0.840 ± 0.100	0.771 ± 0.082	0.788 ± 0.079
	MDSD (mm)	20	0.40 ± 0.50	0.96 ± 0.36	0.90 ± 0.31
L Parotid	DSC	20	0.930 ± 0.073	0.878 ± 0.065	0.872 ± 0.067
	HD95 (mm)	20	2.53 ± 3.63	2.45 ± 1.10	2.77 ± 1.79
	Jaccard	20	0.877 ± 0.123	0.788 ± 0.101	0.779 ± 0.104
	MDSD (mm)	20	0.25 ± 0.44	0.65 ± 0.49	0.75 ± 0.44
R Parotid	DSC	20	0.937 ± 0.068	0.868 ± 0.067	0.872 ± 0.063
	HD95 (mm)	20	1.70 ± 1.69	2.67 ± 1.52	2.64 ± 1.47
	Jaccard	20	0.888 ± 0.116	0.773 ± 0.103	0.779 ± 0.099
	MDSD (mm)	20	0.25 ± 0.44	0.77 ± 0.47	0.82 ± 0.43
L Submandibular	DSC	20	0.911 ± 0.101	0.802 ± 0.166	0.801 ± 0.213
	HD95 (mm)	19	1.93 ± 1.96	2.66 ± 1.62	2.52 ± 2.03
	Jaccard	20	0.851 ± 0.158	0.693 ± 0.180	0.704 ± 0.223
	MDSD (mm)	19	0.37 ± 0.50	0.58 ± 0.51	0.58 ± 0.51
R Submandibular	DSC	20	0.921 ± 0.105	0.805 ± 0.127	0.807 ± 0.191
	HD95 (mm)	20	1.94 ± 2.35	3.47 ± 3.29	3.68 ± 5.45
	Jaccard	20	0.868 ± 0.162	0.690 ± 0.156	0.709 ± 0.213
	MDSD (mm)	20	0.27 ± 0.49	0.82 ± 0.54	0.95 ± 1.02
Esophagus	DSC	19	0.404 ± 0.197	0.861 ± 0.054	0.855 ± 0.066
	HD95 (mm)	19	63.47 ± 28.42	1.82 ± 0.67	2.45 ± 2.20
	Jaccard	19	0.277 ± 0.204	0.760 ± 0.088	0.752 ± 0.099
	MDSD (mm)	19	10.01 ± 12.33	0.26 ± 0.45	0.32 ± 0.48
Spinal canal	DSC	19	0.566 ± 0.142	0.855 ± 0.075	0.850 ± 0.106
	HD95 (mm)	19	44.39 ± 31.04	2.83 ± 1.40	2.77 ± 1.56
	Jaccard	19	0.411 ± 0.167	0.754 ± 0.108	0.752 ± 0.150
	MDSD (mm)	19	1.94 ± 0.86	0.50 ± 0.64	0.59 ± 0.72
Lymph node levels	DSC	20	0.842 ± 0.142	0.819 ± 0.127	0.792 ± 0.043
	HD95 (mm)	20	6.49 ± 5.73	8.07 ± 5.97	9.06 ± 2.30
	Jaccard	20	0.750 ± 0.198	0.714 ± 0.194	0.657 ± 0.061
	MDSD (mm)	20	0.51 ± 0.68	0.71 ± 0.56	0.95 ± 0.22

**Fig. 2:**
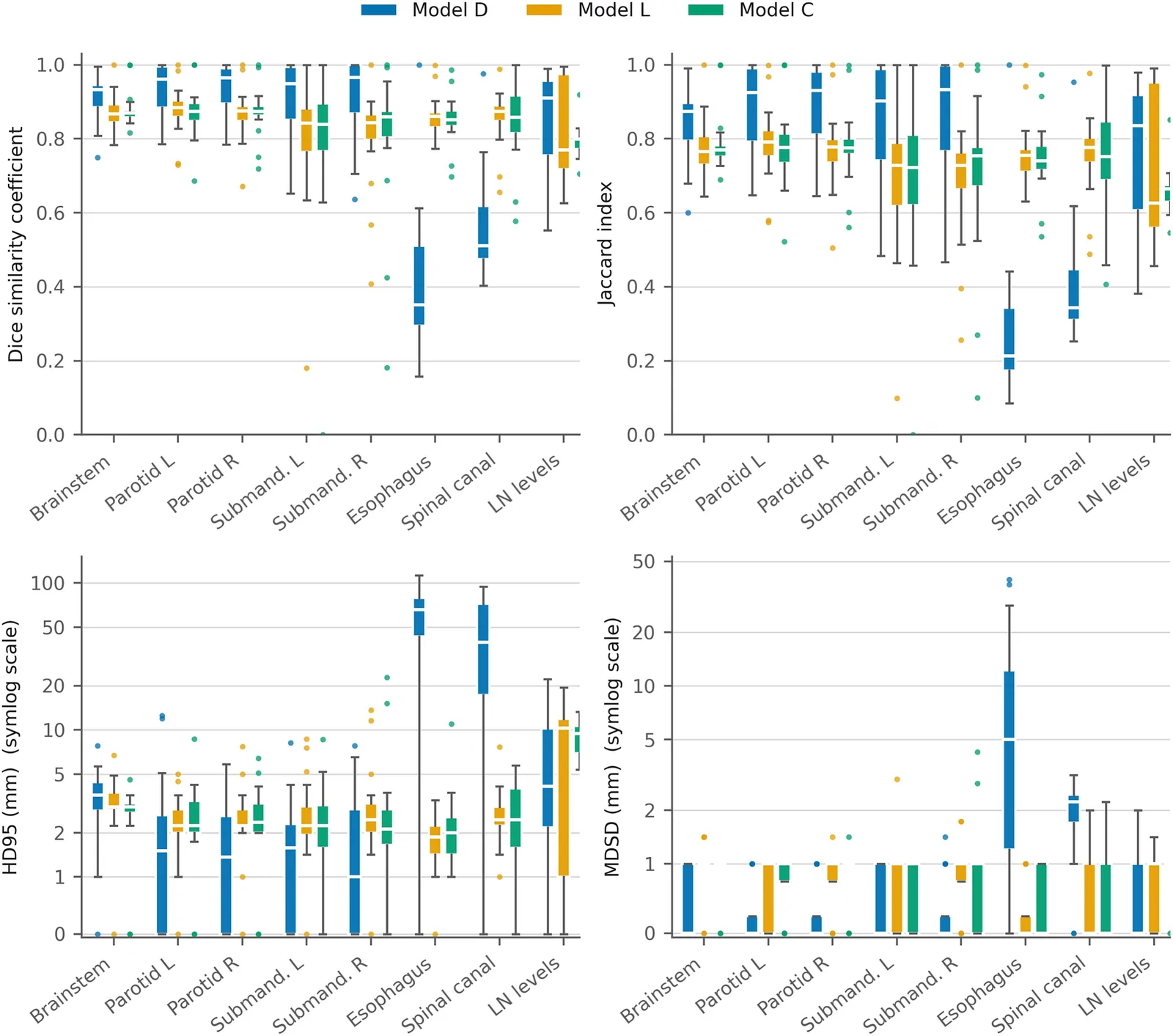
Distribution of the four geometric metrics by structure and model. Box plots show the median, interquartile range and 1.5 × IQR whiskers; points beyond the whiskers are shown individually. HD95 and MDSD use a symmetric-logarithmic scale. Model D shows a markedly wider spread than the other two models.

Directional overlap explains these two values. For the spinal canal, 95% of the Model D contour lies inside the expert contour while covering only 42% of it; for the esophagus the corresponding figures are 81% and 29%. The Model D structures are therefore almost wholly contained within the reference but substantially shorter, at 44% and 35% of the reference volume respectively (Fig. 3).

**Fig. 3:**
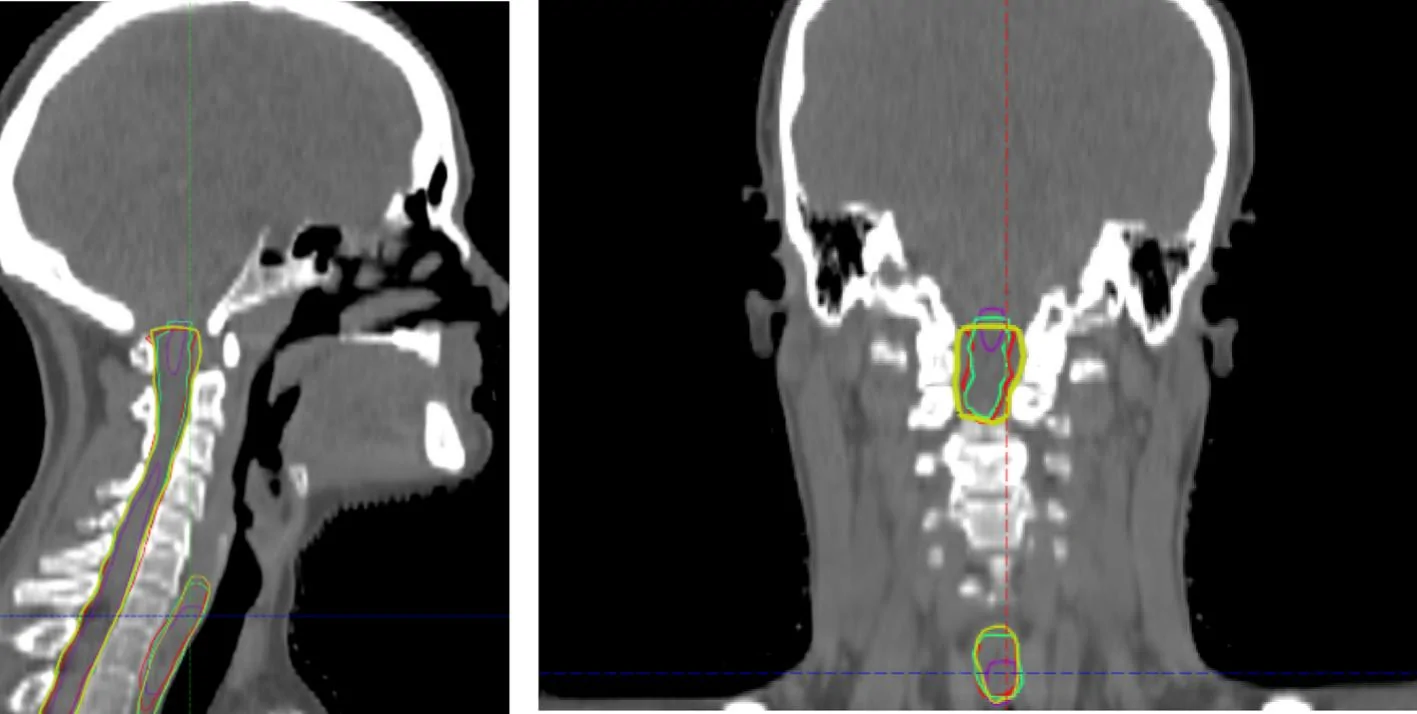
Sagittal (left) and coronal (right) views of the spinal canal and the esophagus, the two structures for which Model D differed most from the expert reference. Expert: magenta; Model L: cyan; Model C: orange; Model D: yellow. (For interpretation of the references to colour in this figure legend, the reader is referred to the web version of this article.)

Across the seven OARs, mean HD95 remained below 3.7 mm for every model and structure except Model D esophagus (63.5 ± 28.4 mm) and Model D spinal canal (44.4 ± 31.0 mm); the merged lymph node region gave higher values for all three models (6.5 to 9.1 mm), reflecting the greater boundary ambiguity of nodal levels. Pooled OAR Jaccard indices were 0.72 ± 0.28 (Model D), 0.75 ± 0.12 (Model L) and 0.75 ± 0.15 (Model C). Mean MDSD was at or below 1.0 mm – that is, within one voxel - for every model and OAR except Model D esophagus (10.0 ± 12.3 mm) and Model D spinal canal (1.9 ± 0.9 mm). Full descriptive statistics are given in Table 1 and Fig. 2 and the corresponding *p*-values in Table 2 and Supplementary Fig. S2; structure volumes are given in Supplementary Table S2.

**Table 2:** *p*-values for all four metrics. Friedman omnibus test across the three models, followed by pairwise Wilcoxon signed-rank tests with Holm-Bonferroni correction within each structure and metric. Descriptive statistics for the same comparisons are given in Table 1.

Structure	Metric	n	Friedman p	D vs L (p)	D vs C (p)	L vs C (p)
Brainstem	DSC	20	0.022	0.098	0.211	0.546
	HD95 (mm)	20	0.060	0.751	0.180	0.751
	Jaccard	20	0.022	0.120	0.165	0.571
	MDSD (mm)	20	0.001	0.023	0.033	0.688
L Parotid	DSC	20	0.047	0.080	0.058	0.674
	HD95 (mm)	20	0.358	1.000	1.000	1.000
	Jaccard	20	0.047	0.072	0.058	0.701
	MDSD (mm)	20	0.004	0.085	0.028	0.562
R Parotid	DSC	20	0.035	0.043	0.036	0.546
	HD95 (mm)	20	0.109	0.219	0.283	0.985
	Jaccard	20	0.035	0.048	0.036	0.498
	MDSD (mm)	20	0.001	0.045	0.045	1.000
L Submandibular	DSC	20	0.157	0.072	0.179	0.784
	HD95 (mm)	19	0.546	0.987	0.987	0.987
	Jaccard	20	0.157	0.058	0.139	0.571
	MDSD (mm)	19	0.292	1.000	1.000	1.000
R Submandibular	DSC	20	0.008	0.064	0.165	0.756
	HD95 (mm)	20	0.106	0.774	1.000	1.000
	Jaccard	20	0.008	0.025	0.127	0.674
	MDSD (mm)	20	0.025	0.052	0.068	0.438
Esophagus	DSC	19	<0.001	<0.001	<0.001	0.798
	HD95 (mm)	19	<0.001	<0.001	<0.001	0.340
	Jaccard	19	<0.001	<0.001	<0.001	0.829
	MDSD (mm)	19	<0.001	<0.001	<0.001	0.812
Spinal canal	DSC	19	<0.001	<0.001	<0.001	0.595
	HD95 (mm)	19	<0.001	<0.001	<0.001	0.702
	Jaccard	19	<0.001	0.002	0.002	0.709
	MDSD (mm)	19	<0.001	0.003	0.006	0.438
Lymph node levels	DSC	20	0.350	1.000	0.160	1.000
	HD95 (mm)	20	0.449	1.000	0.269	1.000
	Jaccard	20	0.350	1.000	0.109	1.000
	MDSD (mm)	20	0.074	0.452	0.124	0.320

Two structures could not be evaluated in all twenty patients. The expert spinal canal contour was unavailable in one patient and Model D produced no esophagus contour in another, so both structures were analyzed in nineteen patients. In one further patient Model C returned an empty left submandibular gland (model volume 0.0 cm3 against an expert volume of 1.6 cm3); this contour is retained in the overlap analysis, where it scores DSC = 0, but is necessarily excluded from HD95 and MDSD, which are undefined for an empty structure.

After Holm-Bonferroni correction the differences between models were considerably more limited than the mean values alone suggest (Table 2). For the esophagus and the spinal canal, Model D differed from both other models on all four metrics (all corrected *p* ≤ 0.006). Elsewhere the picture is sparse, and every surviving comparison lies in the 0.02 to 0.05 band that we treat as exploratory. The right parotid gland was the only structure at which Model D exceeded both other models on an overlap metric (DSC and Jaccard, corrected p 0.036 to 0.048). The right submandibular gland gave one surviving comparison, Model D against Model L on the Jaccard index (*p* = 0.025), but the corresponding DSC comparison was not significant (*p* = 0.064); since DSC and the Jaccard index carry the same information, a difference appearing on only one of them is not treated here as a finding. On MDSD, Model D was closer to the reference than both other models for the brainstem and the right parotid gland and then Model C for the left parotid gland (all *p* ≤ 0.045). The brainstem and the right submandibular gland gave a significant Friedman result with no surviving pairwise overlap comparison for the brainstem; the left submandibular gland was not significant on any overlap metric. Models L and C did not differ on any structure or metric (smallest corrected *p* = 0.32). Pairwise tests were computed for every structure and metric irrespective of the Friedman result, so the pairwise columns of Table 2 should be read together with the omnibus column.

n = number of patients contributing to each row: 20 throughout, except the esophagus and the spinal canal (19, see Methods) and the left submandibular gland for HD95 and MDSD (19, because Model C returned an empty structure in one patient, for which surface distances are undefined).

### Lymph node levels

Over the merged lymph node region, mean DSC was 0.84 for Model D (range 0.55 to 0.99), 0.82 for Model L (0.63 to 0.99) and 0.79 for Model C (0.71 to 0.92); despite the lowest mean, Model C had much the narrowest spread (SD 0.04 against 0.14 and 0.13). Mean HD95 was 6.49, 8.07 and 9.06 mm and mean Jaccard 0.75, 0.71 and 0.66 (Table 1).

Friedman tests revealed no statistically significant overall difference among the three models for lymph node levels on DSC (*p* = 0.35), HD95 (*p* = 0.45), Jaccard index (*p* = 0.35) or MDSD (*p* = 0.074), and unpaired Wilcoxon comparison reached significance after Holm-Bonferroni correction. Volume analysis, by contrast, separated the models clearly: Model D under-estimated the expert volume by a mean of 33.4 cm3 (95% limits of agreement −95.9 to +29.0 cm3, *p* < 0.001) and Model L over-estimated it by 30.5 cm3 (−21.0 to +82.1 cm3, *p* < 0.001), whereas Model C showed no significant deviation (+3.8 cm3, −46.5 to +54.1 cm3, *p* = 0.70). The regression slopes were similar for all three models (0.98, 0.91 and 0.89 for Models D, L and C) and do not distinguish them: it is the offset, not the slope, that differs, and the wide limits of agreement mean that the absence of a systematic bias in Model C should not be read as close per-patient agreement. Comparable overlap at different volumes reflects different geometric strategies: Model L compensates for over-contouring with high coverage of the expert structure, while Model C achieves a similar overlap at a matched volume (Fig. 4).

**Fig. 4:**
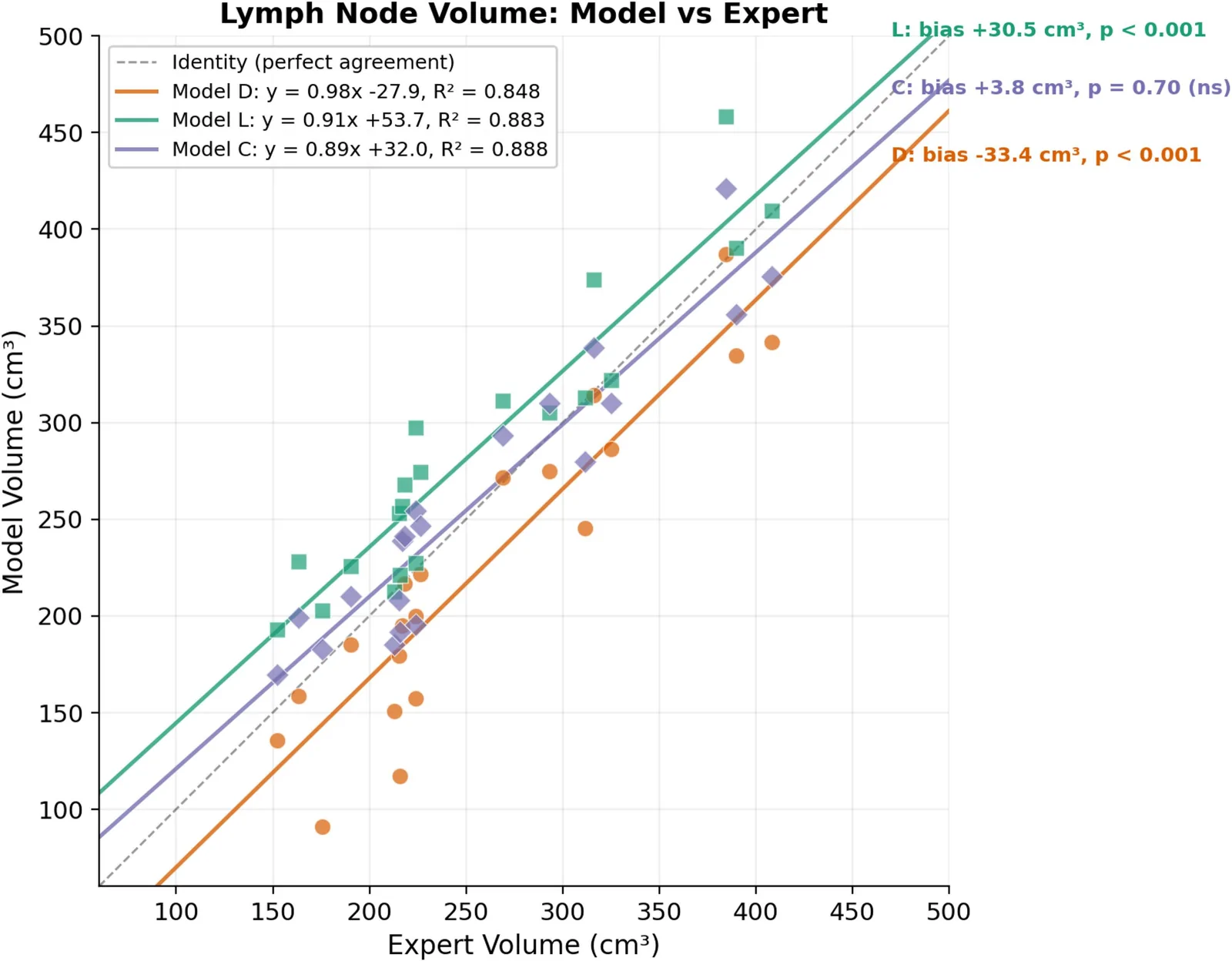
Agreement between model-generated and expert lymph node volumes for the twenty patients. Each point is one patient; the dashed line is the identity line and the solid lines are the least-squares regressions.

This picture changes when the analysis is restricted to the seventeen patients whose lymph node reference contour is independent of all three models (Supplementary Table S3). All three contaminated lymph node references belong to Model L and removing them makes the omnibus test significant for DSC and the Jaccard index (Friedman *p* = 0.035 for both, against 0.350 in the primary analysis) and for MDSD (*p* = 0.030 against 0.074), with Model D highest on every metric (DSC 0.855 against 0.789 for Model L and 0.791 for Model C). No pairwise comparison survives correction in that subset (smallest corrected *p* = 0.060, Model D against Model C on the Jaccard index), so the models still cannot be separated pairwise; but the statement that no overlap metric distinguishes them at all holds only in the primary analysis. Since the sensitivity analysis uses the cleaner reference set, the lymph node overlap results should be regarded as favouring Model D rather than as showing equivalence.

### Subjective assessment

Table 3 reports, for each structure, model and observer, the number of the twenty contours assigned to each category. Converting the scale to a numerical score (a = 1, b = 2, c = 3), the mean modification score pooled over the seven OARs and both observers was 2.03 for Model D, 2.44 for Model L and 2.43 for Model C. The difference was driven mainly by the proportion of contours judged to require major corrections: 25.0% (70/280) for Model D against 6.4% (18/280) for Model L and 5.4% (15/280) for Model C. Contours accepted without any modification represented 27.9% (78/280), 50.0% (140/280) and 48.6% (136/280) respectively. Most modifications were required for the esophagus, for which Model D scored 1.45 (dosimetrist) and 1.35 (radiation oncologist), the lowest scores recorded in the study.

**Table 3:** Subjective assessment: number of the twenty contours in each category, by structure, model and observer.

Structure	Model	Dosimetrist			Radiation oncologist		
		**Major**	**Minor**	**Accept.**	**Major**	**Minor**	**Accept.**
Brainstem	Model D	1*	17	2	0	7	13
	Model L	8*	12	0	0	9	11
	Model C	1*	18	1	0	3	17
Submandibular gland L	Model D	2*	15	3	1*	1	18
	Model L	8*	12	0	0	15	5
	Model C	0	19	1	1*	1	18
Submandibular gland R	Model D	0	20	0	1*	2	17
	Model L	5*	15	0	0	1	19
	Model C	2*	18	0	2*	1	17
Esophagus	Model D	6*	11	3	0	2	18
	Model L	11*	9	0	14*	5	1
	Model C	4*	14	2	2*	9	9
Parotid gland L	Model D	3*	16	1	0	0	20
	Model L	7*	11	2	0	0	20
	Model C	2*	8	10	0	1	19
Parotid gland R	Model D	4*	15	1	0	7	13
	Model L	7*	13	0	0	9	11
	Model C	0	19	1	0	11	9
Spinal canal	Model D	0	7	13	0	2	18
	Model L	2*	15	3	8*	6	6
	Model C	1*	4	15	0	3	17

Bold values marked with an asterisk (*) denote structure-model-observer combinations for which at least one contour required major corrections.

The two observers differed systematically: the dosimetrist was consistently more stringent than the radiation oncologist, with mean scores of 1.69, 2.05 and 2.14 for Models D, L and C against 2.36, 2.82 and 2.72. Both ranked Model D last overall, but not the other two in the same order: the dosimetrist scored Model C above Model L (2.14 against 2.05) and the radiation oncologist the reverse (2.82 against 2.72). Model D scored worst on six of the seven OARs; on the right submandibular gland Model C was marginally the worst (2.33 against 2.35 for Model D and 2.40 for Model L). The subjective ranking does not follow the geometric one: Model D is the most accurate model on the five OARs where the three vendors share a structure definition yet is scored worst subjectively. The subjective scale, unlike the geometric metrics, penalises a contour for being the wrong structure rather than an imprecise version of the right one, which is what the esophagus and spinal canal results represent. Representative axial slices comparing the expert reference with the three automatically generated contour sets for the same patient are shown in Fig. 5.

**Fig. 5:**
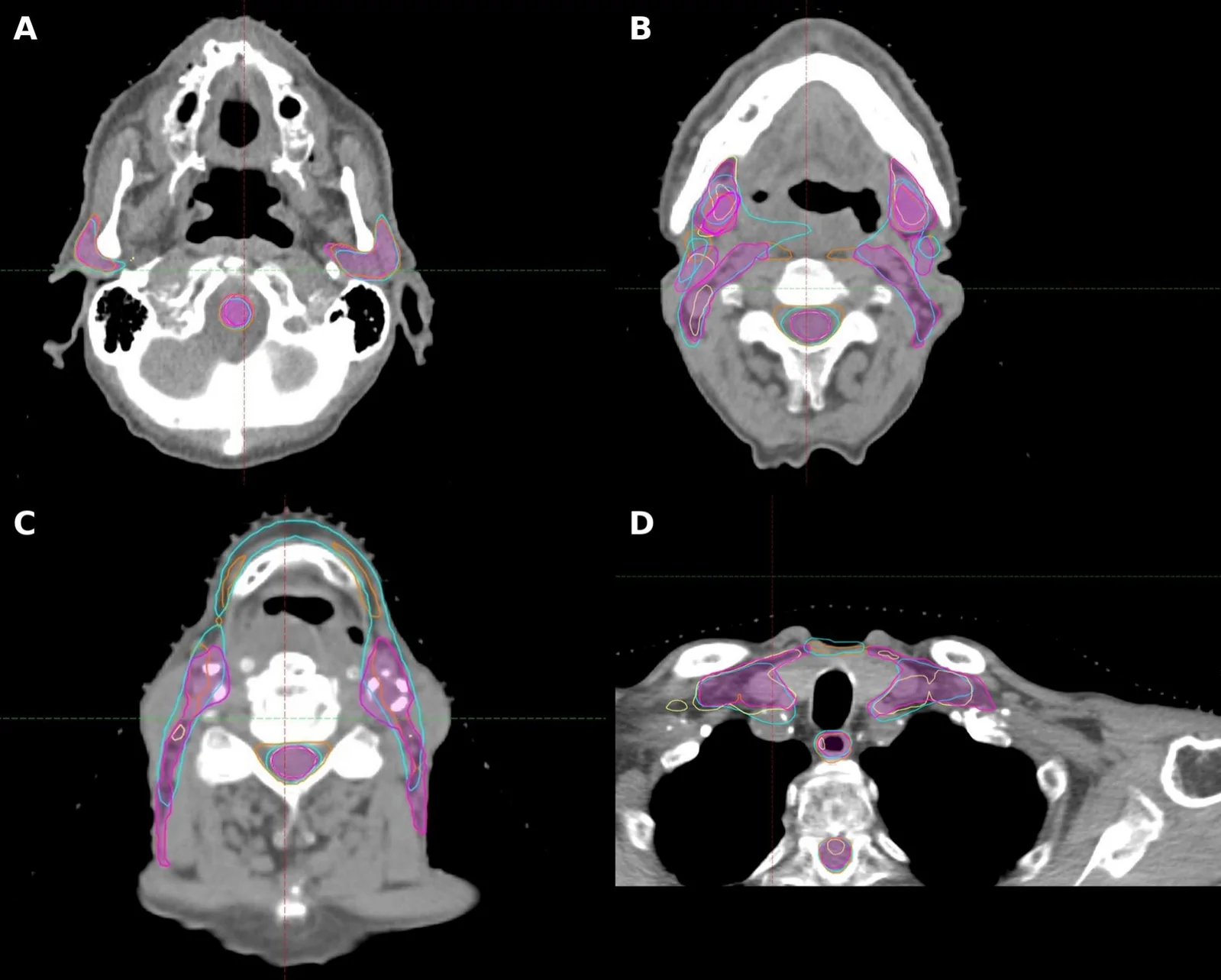
Representative axial slices from one patient, shown from superior to inferior: (A) skull base, (B) oropharynx at the level of the parotid glands, (C) larynx, (D) supraclavicular fossa. The expert reference contours are shown as shaded fills and the three model contours as overlapping solid lines. Expert: magenta; Model L: cyan; Model C: orange; Model D: yellow. (For interpretation of the references to colour in this figure legend, the reader is referred to the web version of this article.)

### Time saving

Manual contouring averaged 28 min for OARs and 16 min for lymph node levels, that is 44 min per patient in total. AI assisted review took approximately 5 min per patient in total. Per-structure contouring times are given in Supplementary Table S1.

## Discussion

To the best of our knowledge, our assessment is among the first single institution studies to benchmark three commercially available DLC models on the same patient cohort for both OAR and lymph node level contouring in the head and neck. Previous multi-vendor comparisons have focused on OARs alone [19], or on single platforms [24], [25].

Head and neck cancer management is one of the most challenging areas in radiation therapy. The precise definition of relatively small OARs, lymph nodes, and target volumes is a key factor in determining the feasibility and effectiveness of radiotherapy treatments. The evaluation of these three models on a single institutional cohort serves two purposes: it provides the quantitative basis on which our department selected and commissioned a DLC solution for routine head and neck contouring, and it adds vendor-comparative data for cervical lymph node levels, which are under-represented in the published literature.

For the five OARs on which all three vendors use the same anatomical definition – brainstem, both parotid glands and both submandibular glands - all three models achieved a mean DSC above 0.80, with the highest agreement for the parotid glands (DSC 0.87 to 0.94, mean HD95 below 2.8 mm) and the brainstem (mean HD95 below 3.7 mm). Jaccard indices followed the same pattern. On these five structures Model D was the most accurate model, although after correction for multiple comparisons the advantage was statistically significant only for the right parotid gland on overlap metrics, and more consistently on MDSD.

The absolute values are in line with published multi-vendor evaluations of head and neck autocontouring, which report mean HD95 in the region of 2 to 5 mm for the parotid glands and the brainstem; our values of 1.7 to 3.7 mm for these structures sit at the favourable end of that range, and the lymph node HD95 of 6.5 to 9.1 mm reflects the greater boundary ambiguity of nodal levels rather than a failure of any model. [19]

The containment pattern reported above explains these two values, and the directional overlap makes the reason plain (Fig. 3). For the spinal canal, 95% of the Model D contour lies inside the expert contour while covering only 42% of it; for the esophagus the corresponding figures are 81% and 29%. Model D is therefore contouring a structure that is almost wholly contained within the reference but substantially shorter, at 44% and 35% of the reference volume respectively. A model segmenting the same structure poorly would place voxels outside the reference and would not show this containment; near-complete containment with low coverage is instead the signature of the spinal cord rather than the spinal canal being contoured, and of a shorter cranio-caudal extent for the esophagus. Model L is close to symmetric on both structures (spinal canal 85% containment with 88% coverage; esophagus 89% with 83%), whereas Model C is asymmetric in the opposite direction (spinal canal 77% containment with 96% coverage), as set out below. This is a difference in structure definition between the vendor and the ESTRO convention used for the reference, not a failure of segmentation. Van Rooij et al. similarly identified the esophagus as one of the lowest performing OARs (DSC 0.60) in deep learning based head and neck. [26] Podobnik et al., comparing three commercial platforms, had to truncate the automatically generated brainstem and spinal cord contours before analysis precisely because their cranio-caudal limits differed from the curated reference, which is the same class of convention difference reported here. [19]

Model C shows a systematic mismatch of its own on the same structure, in the opposite direction and of smaller magnitude. Its spinal canal volume exceeds the expert volume by a mean of 10.6 cm3 (52.8 against 42.2 cm3, ratio 1.31, range 1.00 to 2.33, *p* < 0.001), and while it recovers 96% of the expert contour, 23% of the Model C volume lies outside it. This did not depress its DSC to the same degree because the excess is distributed around a structure that is large relative to the discrepancy, but it is the same class of convention difference and a department adopting Model C would need to check the canal definition in the same way.

More broadly, all three models show statistically significant volume deviations from the expert reference on about half the structures (Supplementary Table S2): five of eight for Model D, four for Model L and four for Model C. Model C over-contours the brainstem by 2.1 cm^3^ (*p* < 0.001) and both parotid glands by about 1 cm^3^ (*p* = 0.009 and *p* = 0.011) in addition to the spinal canal. These deviations are small in absolute terms, but they mean that Model C's freedom from systematic bias is specific to the lymph node region and should not be read as a general property of the model.

For lymph node levels, no overlap metric separated the three models in the primary analysis (mean DSC 0.79 to 0.84, all corrected *p* > 0.05). That equality is partly an artefact of the reference set: restricted to independent references the omnibus test becomes significant with Model D highest (Supplementary Table S3), so Model D is if anything the better model on overlap. Model D also had the widest spread and Model C much the narrowest (SD 0.04 against 0.14 and 0.13). The clearest difference between the models was volumetric: Model C was the only model whose lymph node volumes showed no systematic difference from the expert reference, while Model D under-estimated and Model L over-estimated it by roughly 30 cm^3^ (both *p* < 0.001). The 95% limits of agreement are nonetheless wide for all three models (about ±50 cm^3^ for Model C), so this is an absence of systematic bias rather than close per-patient agreement, and the volume comparisons were not corrected for multiplicity. For a department that has to plan on the output, predictable volume may still matter more than a marginally higher mean overlap.

Research on CT based segmentation of head and neck lymph node levels across multiple vendors remains scarce, which motivated the inclusion of lymph node evaluation alongside the more commonly studied OARs. This study constitutes an exercise of local external validation, evaluating three commercial DLC models on our institutional patient cohort.

As demonstrated in our previous research on atlas-based methods (M-ABAS), automation significantly reduced contouring time for head and neck OARs compared to both manual and semi-automated approaches, with additional efficiency gains as the atlas library expanded [27].

The present results confirm that DLC delivers a further change in step, with review times reduced to approximately 5 min per region. Numerous studies have reported similar time savings in the head and neck region [24], [25], [28].

In our department, manual delineation of the structures evaluated here took 44 min per patient on average (28 min for the seven OARs and 16 min for the lymph node levels), of which the 28 min spent on the seven OARs is consistent with the 20 to 40 min per patient reported in the literature for OAR delineation alone. Reducing this to approximately 5 min of review per region changes the nature of the task: starting from a high-quality automated draft, the dosimetrist or the experienced radiation therapist moves from creating contours to editing them, which reduces repetitive strain and cognitive fatigue in addition to saving time.

The integration of DLC into head and neck treatment planning improves the efficiency of the contouring workflow without a loss of geometric agreement with expert contours for the structures evaluated here. The practical benefit for our department is twofold: a reduction in the time spent on delineation, and a reduction in the between-observer variation that arises when several staff members contour the same structures. The present dataset, comprising twenty patients with expert validated contours of seven OARs and five lymph node levels, is not large enough to serve as a general benchmark, but it is sufficient to support local commissioning and to define the acceptance thresholds and quality assurance checks that we apply to automatically generated contours before clinical use.

### Limitations

Four limitations bear directly on how these results should be read. First, and most important, part of the expert reference set is not independent of the models under evaluation: in 49 of 476 structure-patient comparisons the reference contour is voxel-identical to one model's output. Those 49 comparisons are spread over seventeen of the twenty patients and fall unevenly across models (Model D 28 structures, Model C 12, Model L 9). How this arose could not be established retrospectively; either way the model is compared with itself and its score for that structure is inflated by construction. The sensitivity analysis in Supplementary Table S3 shows that the esophagus and spinal canal findings are unaffected and that the brainstem MDSD difference strengthens, that the right parotid advantage survives only against Model C, that the left parotid and right submandibular differences do not survive at all, and that the lymph node omnibus test – non-significant on the full set - becomes significant in favour of Model D. The filter is not neutral between models: it removes 28 Model D structures, 12 Model C and 9 Model L, and it reduces the subsets to between ten and eighteen patients, so some differences disappear through loss of power rather than through removal of contamination. The effect sizes for the parotid glands, for instance, are essentially unchanged in the subset while the *p*-values rise. Those differences should therefore be regarded as unconfirmed, and a reference set delineated independently of all candidate models would be required to settle them. Second, the subjective assessment inherits the same problem: the radiation oncologist who scored the contours is the same person who produced the reference, so for the structures where a model's contour had been accepted into the reference, he was scoring a contour he had already accepted. Since 28 of the 49 such structures belong to Model D, this may bias the subjective comparison, and agreement between the two observers was in any case not quantified formally. Third, the sample of 20 patients limits statistical power, particularly for the smaller structures, and the reference was produced by a single expert without a consensus step; correction for multiple comparisons was applied within each structure and metric but not across them. Non-significant pairwise differences are reported as an absence of evidence and are not evidence of equivalence; in particular, the statement that Models L and C could not be distinguished rests on non-significance at this sample size and is not a demonstration that they perform identically. The volume comparisons were not corrected for multiple testing, whereas the geometric comparisons were. Fourth, no dosimetric analysis was performed, so the clinical impact of the volumetric differences reported here remains unquantified. The restriction to the OAR set common to all three platforms also excluded structures available in individual models, lymph node levels I to V were analyzed only as a merged region, and the three vendors neither disclose their training data to the same level of detail nor follow identical delineation conventions - a difference that is the most likely explanation for the esophagus and spinal canal results rather than any failure of segmentation quality. Future work should use an independent multi-observer reference, add dosimetric impact analysis, and draw on larger multi-institutional cohorts.

## Conclusion

For the great majority of structures, the three DLC models produced contours of clinically usable quality, with substantial time savings over manual contouring. The exceptions are Model D on the esophagus and the spinal canal, which fall below the DSC threshold used here and were scored as requiring major corrections in most patients, and which reflect a difference in structure definition rather than a segmentation failure.

Taken together, the geometric, volumetric and subjective results support three conclusions. Models L and C could not be distinguished on any metric or structure. Model D differs from both on the esophagus and the spinal canal, where the directional overlap and the sagittal views in Fig. 3 indicate a difference in structure definition rather than in segmentation quality, so a department adopting Model D would need to align those two definitions locally; Model C shows a smaller mismatch of the same kind on the spinal canal. For lymph node levels, Model D achieved the highest mean overlap and the lowest HD95, and was ahead on the independent-reference subset, while Model C gave the narrowest spread and the only volumes without systematic bias, though with limits of agreement as wide as the other models. The choice between models therefore depends on whether a department values peak geometric accuracy or volumetric predictability. Because part of the reference set was not independent of the models, the differences between Model D and the other two on the parotid and submandibular glands require confirmation against an independent reference.

Our study demonstrates how the use of commercially available deep learning contour models can efficiently supplement the contouring process of head and neck organs at risk (OAR) and lymph node contours in terms of reducing the time spent by the radiation oncologist and dosimetrist for patients undergoing treatment for hypopharyngeal/laryngeal tumors.

## Generative AI use

Not applicable.

## CRediT authorship contribution statement

**Adam Miovecz:** Writing – original draft, Methodology, Investigation, Formal analysis, Data curation, Conceptualization. **Bettina Nagy:** Validation. **Daniel Gugyeras:** Validation, Investigation. **Daniel Fajtai:** Visualization, Software. **David Sipos:** Methodology. **Csaba Vandulek:** Writing – review & editing. **Arpad Kovacs:** Writing – review & editing, Supervision. **Ferenc Lakosi:** Writing – review & editing, Validation, Supervision.

## Ethics approval and consent to participate

The present retrospective study was approved and conducted in accordance with the institutional research and ethics guidelines (Kaposi Mór Teaching Hospital, IG/00547–0000/2024) and with the Helsinki Declaraion of 1975 and 1989. Furthermore, prior to analysis, all patient data were fully anonymized in accordance with the Hungarian GDPR regulation (Act XLVII of 1997 on the Processing and Protection of Health Data and Related Personal Data) and the GDPR Regulation (EU) 2016/679.

## Ethics declaration

The authors have NOT obtained informed consent from participants or their legal representatives. The requirement for informed consent was waived by the Regional Research Ethics Committee, Clinical Centre, University of Pécs, Hungary (approval no. 135-PTERKEB2026/1), on the grounds that the study was retrospective and non-interventional, in accordance with Hungarian Government Decree 235/2009 (X.20.) Section 17/A (1) c-d). The study used previously acquired routine planning CT images and contours; no additional procedures were performed on patients. All data were fully anonymised before analysis, and no identifiable patient information is presented in this article.

This study was performed in compliance with relevant laws, regulatory frameworks and guidelines where the research took place. This study was approved by the Regional Research Ethics Committee, Clinical Centre, University of Pécs (Pécsi Tudományegyetem Klinikai Központ, Regionális Kutatásetikai Bizottság), Pécs, Hungary (Approval No. 135-PTERKEB2026/1)

## Funding

The authors have no funding sources to declare.

## Declaration of competing interest

The authors declare that they have no competing interests.

## Data Availability

Data required for this study may be made available by the author(s) upon reasonable request.
