# Supplementary material for: Clinical evaluation of three deep learning contouring models for head and neck organs at risk and lymph node levels
Source: Tech Innov Patient Support Radiat Oncol. 2026 Sep 23;40:100437. doi: 10.1016/j.tipsro.2026.100437 (PMC13635919; doi:10.1016/j.tipsro.2026.100437)
Supplement: Supplementary file 1 [file mmc1.docx]

Supplementary material

Deep learning contouring of head and neck organs at risk and cervical lymph node levels: a three-vendor comparison. All values were recomputed from the per-patient metric export; the analysis scripts are provided alongside this file.

## Supplementary Table S1. Contouring and review times per structure

Table S1. Mean manual delineation time and mean AI-assisted review time per structure. Manual times were measured per region with a stopwatch during delineation. AI-assisted review was timed as a single total per patient; the per-region and per-structure figures are an indicative breakdown of that total.

| **Structure** | **Manual delineation (min)** | **AI-assisted review (min)** |
| --- | --- | --- |
| Brainstem | 4 | 0.42 |
| Left parotid gland | 5 | 0.58 |
| Right parotid gland | 5 | 0.58 |
| Left submandibular gland | 3 | 0.21 |
| Right submandibular gland | 3 | 0.21 |
| Esophagus | 5 | 0.75 |
| Spinal canal | 3 | 0.25 |
| OAR sub-total | 28 | 3 |
| Lymph node levels I-V | 16 | 2 |
| Lymph node sub-total | 16 | 2 |

## Supplementary Table S2. Volume agreement with the expert reference

Table S2. Structure volumes and their agreement with the expert reference.

| **Structure** | **Model** | **n** | **Expert volume (cm3)** | **Model volume (cm3)** | **Mean difference (cm3)** | **95% limits of agreement** | **p** |
| --- | --- | --- | --- | --- | --- | --- | --- |
| Brainstem | D | 20 | 24.5 | 21.8 | -2.7 | [-6.6, +1.2] | <0.001 |
| Brainstem | L | 20 | 24.5 | 22.7 | -1.8 | [-5.9, +2.3] | <0.001 |
| Brainstem | C | 20 | 24.5 | 26.6 | +2.1 | [-1.8, +6.0] | <0.001 |
| L Parotid | D | 20 | 21.4 | 22.5 | +1.1 | [-2.6, +4.8] | 0.012 |
| L Parotid | L | 20 | 21.4 | 21.7 | +0.3 | [-2.9, +3.5] | 0.648 |
| L Parotid | C | 20 | 21.4 | 22.4 | +1.0 | [-2.6, +4.5] | 0.009 |
| R Parotid | D | 20 | 21.0 | 21.4 | +0.4 | [-3.7, +4.4] | 0.241 |
| R Parotid | L | 20 | 21.0 | 21.8 | +0.8 | [-3.7, +5.2] | 0.105 |
| R Parotid | C | 20 | 21.0 | 22.1 | +1.1 | [-2.8, +5.0] | 0.011 |
| L Submandibular | D | 20 | 7.0 | 7.2 | +0.1 | [-1.8, +2.1] | 0.622 |
| L Submandibular | L | 20 | 7.0 | 6.8 | -0.2 | [-2.9, +2.5] | 0.674 |
| L Submandibular | C | 20 | 7.0 | 6.3 | -0.7 | [-3.9, +2.5] | 0.096 |
| R Submandibular | D | 20 | 6.4 | 6.1 | -0.3 | [-2.0, +1.4] | 0.738 |
| R Submandibular | L | 20 | 6.4 | 6.9 | +0.4 | [-1.6, +2.5] | 0.027 |
| R Submandibular | C | 20 | 6.4 | 6.4 | -0.1 | [-3.0, +2.8] | 0.293 |
| Esophagus | D | 19 | 14.8 | 5.3 | -9.5 | [-19.1, +0.1] | <0.001 |
| Esophagus | L | 20 | 14.8 | 13.6 | -1.1 | [-4.0, +1.7] | 0.001 |
| Esophagus | C | 20 | 14.8 | 14.3 | -0.4 | [-3.5, +2.7] | 0.097 |
| Spinal canal | D | 19 | 42.2 | 17.4 | -24.8 | [-44.3, -5.3] | <0.001 |
| Spinal canal | L | 19 | 42.2 | 43.1 | +0.9 | [-16.4, +18.3] | 1.000 |
| Spinal canal | C | 19 | 42.2 | 52.8 | +10.6 | [-7.4, +28.6] | <0.001 |
| Lymph node levels | D | 20 | 256.6 | 223.2 | -33.4 | [-95.9, +29.0] | <0.001 |
| Lymph node levels | L | 20 | 256.6 | 287.1 | +30.5 | [-21.0, +82.1] | <0.001 |
| Lymph node levels | C | 20 | 256.6 | 260.4 | +3.8 | [-46.5, +54.1] | 0.701 |

*Mean difference is model minus expert. Limits of agreement are mean difference +/- 1.96 SD of the differences. p from the Wilcoxon signed-rank test of the per-patient difference against zero. The esophagus and spinal canal rows for Model D show substantial under-contouring (35 % and 44 % of the expert volume). The volume ratio alone is not tight enough to prove a definition difference (coefficient of variation 0.61 for the esophagus, 0.37 for the spinal canal); the directional overlap is the stronger evidence, since 81 % and 95 % of the Model D volume lies inside the expert contour while covering only 29 % and 42 % of it. Model C over-contours the spinal canal in the opposite direction (ratio 1.31).*

## Supplementary Table S3. Sensitivity analysis: independent reference contours only

Table S3. Primary analysis (all patients) beside the sensitivity analysis restricted to patients whose expert contour for that structure is independent of all three models.

| **Structure** | **Metric** | **n (all)** | **Friedman p (all)** | **n (independent)** | **Friedman p (independent)** | **D vs L** | **D vs C** | **L vs C** |
| --- | --- | --- | --- | --- | --- | --- | --- | --- |
| Brainstem | DSC | 20 | 0.022 | 15 | 0.017 | 0.096 | 0.077 | 0.890 |
| Brainstem | HD95 (mm) | 20 | 0.060 | 15 | 0.029 | 0.271 | 0.051 | 0.700 |
| Brainstem | Jaccard | 20 | 0.022 | 15 | 0.017 | 0.096 | 0.054 | 0.934 |
| Brainstem | MDSD (mm) | 20 | 0.001 | 15 | <0.001 | 0.012 | 0.006 | 0.875 |
| L Parotid | DSC | 20 | 0.047 | 10 | 0.301 | 0.750 | 0.480 | 0.750 |
| L Parotid | HD95 (mm) | 20 | 0.358 | 10 | 0.656 | 1.000 | 1.000 | 0.586 |
| L Parotid | Jaccard | 20 | 0.047 | 10 | 0.301 | 0.645 | 0.480 | 0.695 |
| L Parotid | MDSD (mm) | 20 | 0.004 | 10 | 0.018 | 0.438 | 0.094 | 0.500 |
| R Parotid | DSC | 20 | 0.035 | 12 | 0.039 | 0.085 | 0.048 | 0.850 |
| R Parotid | HD95 (mm) | 20 | 0.109 | 12 | 0.192 | 0.532 | 0.316 | 0.850 |
| R Parotid | Jaccard | 20 | 0.035 | 12 | 0.039 | 0.085 | 0.037 | 0.791 |
| R Parotid | MDSD (mm) | 20 | 0.001 | 12 | 0.003 | 0.078 | 0.059 | 1.000 |
| L Submandibular | DSC | 20 | 0.157 | 10 | 0.150 | 0.480 | 0.480 | 0.695 |
| L Submandibular | HD95 (mm) | 19 | 0.546 | 10 | 0.641 | 1.000 | 1.000 | 1.000 |
| L Submandibular | Jaccard | 20 | 0.157 | 10 | 0.150 | 0.252 | 0.252 | 0.625 |
| L Submandibular | MDSD (mm) | 19 | 0.292 | 10 | 0.041 | 0.375 | 0.375 | 1.000 |
| R Submandibular | DSC | 20 | 0.008 | 10 | 0.027 | 0.645 | 0.645 | 0.393 |
| R Submandibular | HD95 (mm) | 20 | 0.106 | 10 | 0.368 | 1.000 | 1.000 | 0.316 |
| R Submandibular | Jaccard | 20 | 0.008 | 10 | 0.027 | 0.393 | 0.393 | 0.393 |
| R Submandibular | MDSD (mm) | 20 | 0.025 | 10 | 0.045 | 0.047 | 0.148 | 0.750 |
| Esophagus | DSC | 19 | <0.001 | 17 | <0.001 | <0.001 | <0.001 | 0.712 |
| Esophagus | HD95 (mm) | 19 | <0.001 | 17 | <0.001 | <0.001 | <0.001 | 0.898 |
| Esophagus | Jaccard | 19 | <0.001 | 17 | <0.001 | <0.001 | <0.001 | 0.678 |
| Esophagus | MDSD (mm) | 19 | <0.001 | 17 | <0.001 | <0.001 | <0.001 | 1.000 |
| Spinal canal | DSC | 19 | <0.001 | 18 | <0.001 | <0.001 | 0.001 | 0.325 |
| Spinal canal | HD95 (mm) | 19 | <0.001 | 18 | <0.001 | <0.001 | <0.001 | 0.404 |
| Spinal canal | Jaccard | 19 | <0.001 | 18 | <0.001 | 0.003 | 0.003 | 0.417 |
| Spinal canal | MDSD (mm) | 19 | <0.001 | 18 | <0.001 | 0.005 | 0.011 | 0.438 |
| Lymph node levels | DSC | 20 | 0.350 | 17 | 0.035 | 0.808 | 0.104 | 0.808 |
| Lymph node levels | HD95 (mm) | 20 | 0.449 | 17 | 0.056 | 0.449 | 0.191 | 0.449 |
| Lymph node levels | Jaccard | 20 | 0.350 | 17 | 0.035 | 0.808 | 0.060 | 0.808 |
| Lymph node levels | MDSD (mm) | 20 | 0.074 | 17 | 0.030 | 0.380 | 0.124 | 0.578 |

*A reference contour was treated as non-independent when any model reached DSC >= 0.99 against it. In 48 of the 49 such comparisons the HD95 was also exactly 0.0 mm, in all 49 the MDSD was 0.0 mm and in all 49 the volumes agreed to better than 1 %, i.e. the two masks are the same voxels. The pairwise columns are Holm-Bonferroni corrected p-values from the sensitivity analysis. Note that this filter preferentially removes the patients whose contours needed no editing, so the remaining subset is enriched for anatomically difficult cases; the sensitivity analysis is therefore conservative rather than unbiased.*

## Supplementary Table S4. Reference contours identical to a model output, by patient

Table S4. For each patient, the number of structures whose expert reference contour is voxel-identical to one of the three automatically generated contours.

| **Patient** | **Structures identical to a model** | **Model(s)** | **Structures** |
| --- | --- | --- | --- |
| 7_20231107 | 5 | D/L | L Parotid; L Submandibular; Lymph node levels; R Parotid; R Submandibular |
| 8_20230921 | 2 | D | L Parotid; R Parotid |
| 9_20230919 | 0 | - | - |
| 10_20230907 | 1 | D | Brainstem |
| 11_20230824 | 2 | D | L Submandibular; R Submandibular |
| 12_20230615 | 5 | C | Brainstem; L Parotid; L Submandibular; R Parotid; R Submandibular |
| 13_20230516 | 0 | - | - |
| 14_20230509 | 2 | D | L Parotid; R Parotid |
| 15_20230307 | 5 | C | Brainstem; L Parotid; L Submandibular; R Parotid; Spinal canal |
| 16_20230221 | 2 | C | L Submandibular; R Submandibular |
| 17_20230103 | 1 | D | L Parotid |
| 18_20231128 | 1 | L | Esophagus |
| 19_20221213 | 0 | - | - |
| 20_20231221 | 7 | D/L | Brainstem; Esophagus; L Parotid; L Submandibular; Lymph node levels; R Parotid; R Submandibular |
| 21_20231107 | 5 | D | Brainstem; L Parotid; L Submandibular; R Parotid; R Submandibular |
| 22_20231114 | 4 | D | L Parotid; L Submandibular; R Parotid; R Submandibular |
| 23_20231011 | 1 | L | Lymph node levels |
| 24_20231011 | 2 | D | L Submandibular; R Submandibular |
| 25_20231025 | 3 | D | L Parotid; L Submandibular; R Submandibular |
| 26_20230927 | 1 | D | R Submandibular |

*Seventeen of the twenty patients are affected; only patients 9, 13 and 19 have no such structure. In twelve of the seventeen, two or more affected structures fall within a single model, which is consistent with an automatically generated contour having been accepted into the reference set without modification rather than with coincidence.*

## Supplementary Figure S1. Evidence on reference independence

Figure S1. Left: distribution of DSC between each model and the expert reference, pooled over all structures. The spike at DSC ~ 1.0 cannot arise from independent manual delineation. Right: number of structures per patient whose expert contour is voxel-identical to a model contour, coloured by which model.

## Supplementary Figure S2. Pairwise p-value heatmap (formerly Figure 2)

Figure S2. Holm-Bonferroni corrected pairwise p-values for all four metrics and all eight structures, moved from the main text at the suggestion of Reviewer 2 and regenerated from the corrected analysis. Cells are shaded on a logarithmic scale; unshaded cells are p > 0.05. This figure and Table 2 present the same numbers.
